# A Case of Thiazide-Induced Pancreatitis

**DOI:** 10.7759/cureus.98739

**Published:** 2025-12-08

**Authors:** Abdulla Almajed, Shooq Alsebaie, Nora McVinnie, Augusto R Fernandez-Aristi, Jasmine Omar, Megan Dekker

**Affiliations:** 1 Internal Medicine, Henry Ford Health System, Detroit, USA; 2 Cardiology, Mohammed Bin Khalifa Bin Salman Al Khalifa Specialist Cardiac Centre, Awali, BHR

**Keywords:** adverse effect, drug-induced acute pancreatitis, gastroenterology, pancreatitis, thiazide

## Abstract

Acute pancreatitis is the inflammation of the pancreas that is most commonly caused by gallstones or alcohol in adults. Other less likely causes are autoimmune, hypercalcemia, hypertriglyceridemia, scorpion sting, trauma, idiopathic, post-endoscopic retrograde cholangiopancreatography (ERCP), or drug-induced. Our patient is a 72-year-old woman with a past medical history of hypertension (on hydrochlorothiazide 12.5 mg, amlodipine 10 mg, and losartan 100 mg), chronic hypoxic respiratory failure, and previous deep vein thrombosis* *(DVT) (on apixaban 2.5 mg) who presented to the emergency department with complaints of abdominal pain. She was hemodynamically stable with epigastric pain that radiated to the back and was tender on palpation. Work-up revealed a lipase of 9,130 IU/L, normal lipid profile, leukocytosis of 13,500 cells/mm^3^, and negative urinalysis. Imaging computed tomography (CT) of the abdomen shows acute interstitial pancreatitis with no glandular necrosis, no regional venous thrombosis, and no signs of cholelithiasis. Work-up was negative for other causes of acute pancreatitis: no recent history of trauma, no recent scorpion sting, no steroid use, low autoimmune pancreatitis antibodies, normal calcium levels, normal triglycerides, and no recent history of ERCP. CT of the abdomen as an outpatient follow-up after eight weeks revealed no underlying malignancy. On the basis of exclusion, the patient's thiazide diuretic was likely the cause of her acute pancreatitis with no other possible causes.

In clinical presentations with no typical identifiable causes, it is important to have a high index of suspicion of other rare causes. In this case, an accurate medication reconciliation and a high index of suspicion allowed for the identification of this possible but less common cause.

## Introduction

Acute pancreatitis (AP) is an inflammatory process of the pancreas with a wide spectrum of clinical presentations ranging from mild presentations to severe that require in-hospital admission. In the United States, the incidence of AP is four per 1000 people with approximately 130,000 new cases annually and a mortality rate of 3.5% [1]. The most common causes of AP are gallstones and alcohol in the United States. Other less common causes are hypertriglyceridemia, autoimmune diseases, trauma, endoscopic retrograde cholangiopancreatography (ERCP), and medications. Drug-induced pancreatitis is rare and accounts for less than 2% of cases in the United States [2].

There are currently over 500 medications in the literature that have been reported to cause drug-induced pancreatitis [3]. Diagnosis of drug-induced pancreatitis requires the diagnosis of AP initially. If the work-up for other causes of AP is negative and the patient is on a medication that can cause pancreatitis, then the diagnosis of drug-induced pancreatitis can be made [4]. This diagnosis is on the basis of temporal association, but causality is often difficult to establish.

The exact mechanism of drug-induced pancreatitis is not well understood [5], but the management includes supportive care and withdrawal of the offending agent. This requires a strong index of suspicion to identify the causative agent early on, especially in a commonly used medication class to treat hypertension like hydrochlorothiazide. This is important as continuous exposure to the offending agent predisposes the patient to recurrent pancreatitis and its fatal complications.

## Case presentation

A 72-year-old woman of African American descent with a past medical history of hypertension, severe COVID-19 infection complicated by chronic hypoxic respiratory failure on 3 L home oxygen, and previous pulmonary embolism due to deep vein thrombosis (DVT) presented to the emergency department with a complaint of abdominal pain. The abdominal pain started suddenly after she woke up and was localized in the epigastric region with radiation to the back. It was initially waxing and waning for a couple of hours but then remained constant. The patient described the pain as sharp with a severity of 10 out of 10. She was able to tolerate oral intake that day, and it did not exacerbate the pain. It was associated with subjective fever and chills. There was no nausea or vomiting, and she did have a bowel movement. The patient denied dysuria, urinary changes, and bowel changes. She also denied recent alcohol drinking, illicit substance use, trauma to the abdomen, exposure to scorpions, or a recent surgical procedure. The patient is not married and has not had any pregnancies, and her surgical history included a laparoscopic cholecystectomy for symptomatic cholelithiasis. Home medications include albuterol inhaler, amlodipine, apixaban, and losartan-hydrochlorothiazide.

Vital signs on admission were as follows: blood pressure of 148/88 mmHg, heart rate of 84 beats per minute, respiratory rate of 30 breaths per minute, temperature of 36.2°C, oxygen saturation of 100% on 3 L nasal cannula, and blood glucose of 260 mg/dL. On examination, the patient appeared to be in distress with intolerable pain and tachypnea. Respiratory examination revealed bilateral equal air entry, bronchovesicular breath sounds, and no added sounds. Abdominal examination revealed a symmetric non-distended abdomen with laparoscopic scars. There were no Cullen or Grey Turner signs. The abdomen was soft with generalized tenderness that was maximal in the epigastric region with guarding but no rigidity or rebound tenderness. McBurney's and Murphy's signs were not appreciated. There was no organomegaly or palpable masses. Auscultation revealed normoactive bowel sounds and no bruits. There was no lower limb pitting edema or swelling. The initial differential diagnoses were AP, acute appendicitis, acute cholecystitis, nephrolithiasis, and perforation of a duodenal ulcer.

Initial lab investigations in the emergency department included a complete blood count, basic metabolic profile, liver function test, lipid panel, plasma lactate, lipase, and troponin (Table 1). Urine analysis and urine drug screen were also ordered (Table 2). Labs showed leukocytosis of 13,500 cells/mm^3^, neutrophilia with an absolute count of 10.9 K/μL, an elevated lipase of 9,130 IU/L, plasma lactate of 3.3 mmol/L, a normal lipid panel, and a normal liver function panel. Urine analysis was normal, and the drug screen was negative. In view of the clinical presentation, leukocytosis, and elevated lactate, the emergency department activated code sepsis with the initiation of intravenous (IV) empirical antibiotics and fluids while awaiting radiologic investigations.

**Table 1:** Blood laboratory tests ALT: alanine aminotransferase; AST: aspartate aminotransferase

Lab test	Result	Reference range
White blood cell count	13.5 K/μL	3.8-10.6 K/μL
ALT	15 IU/L	<52 IU/L
AST	21 IU/L	<35 IU/L
Bilirubin (total)	0.5 mg/dL	<1.2 mg/dL
Bilirubin (direct)	0.1 mg/dL	<0.3 mg/dL
Alkaline phosphatase	70 IU/L	40-140 IU/L
Lipase	9,130 IU/L	<82 IU/L
Troponin	4 ng/L	<19 ng/L
Lactate (whole blood)	3.3 mmol/L	<2.1 mmol/L

**Table 2:** UA and urine drug screen test UA: urinalysis; HPF: high-power field

Test	Result	Reference range
Urine pH	5	5-8
Protein UA	Negative	Negative
Glucose UA	Negative	Negative
Blood UA	Negative	Negative
Nitrite UA	Negative	Negative
Leukocyte esterase UA	Negative	Negative
White blood cell UA	2/HPF	<10/HPF
Drug screen urine	Negative	Negative

**Figure 1:**
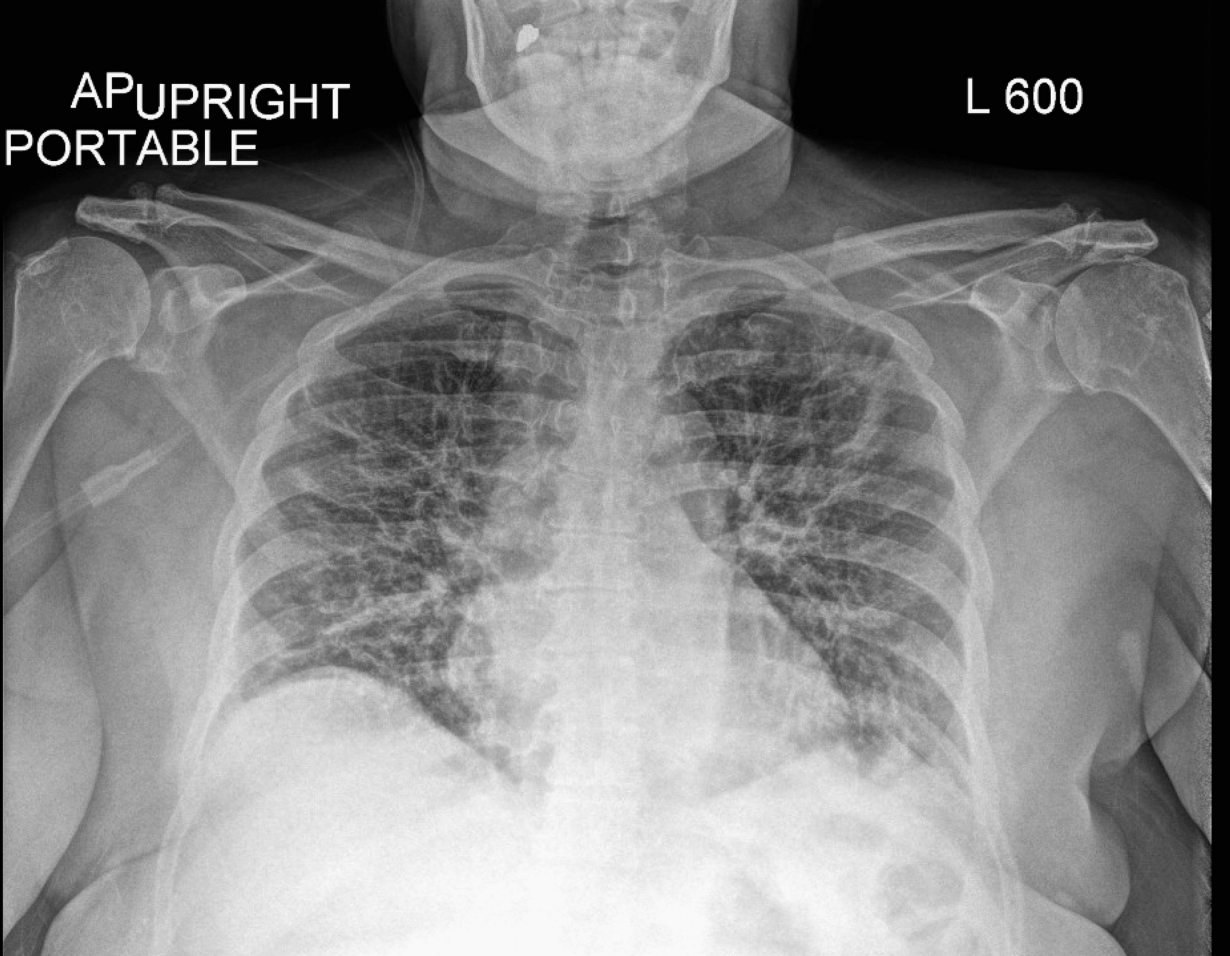
Portable chest X-ray

**Figure 2:**
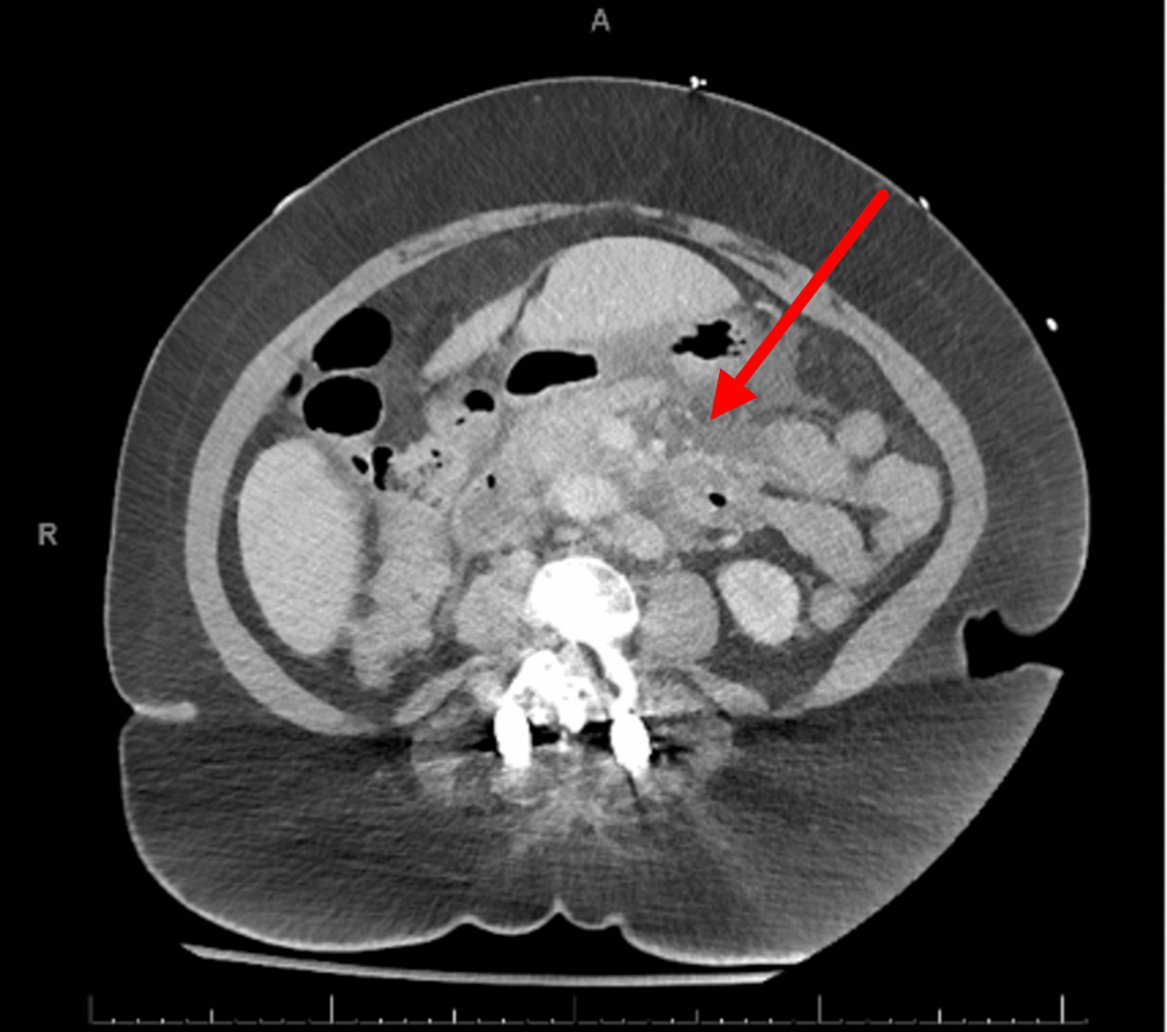
CT of the abdomen CT: computed tomography

A chest X-ray was ordered showing no signs of consolidations, pleural effusions, or cardiomegaly (Figure 1). Computed tomography (CT) of the abdomen revealed findings compatible with acute interstitial pancreatitis (red arrow), no glandular necrosis, no walled-off collections, and no regional venous thrombosis (Figure 2).

The diagnosis of AP was made with the patient meeting all the three criteria of typical abdominal pain, serum lipase higher than the upper normal limit by three times, and characteristic CT findings. The patient was started on continuous IV fluids and kept on a nothing by mouth (NPO) diet and received pain control with admission to the hospital. 

On the general medicine floor, the patient underwent a pancreatitis work-up during her hospital admission with a normal abdominal ultrasound, negative autoimmune antibodies, negative phosphatidylethanol (PEth) test, normal calcium, normal triglycerides, and negative viral panel (Table 3). Gastroenterology was consulted with recommendations to continue supportive treatment, stop hydrochlorothiazide, and follow up as an outpatient with a repeat abdominal CT scan in 6-8 weeks. The patient had an unremarkable hospital course with gradual improvement of abdominal pain and progression of oral diet. The repeat CT scan 6-8 weeks later showed the resolution of the pancreatic inflammation and confirmed the absence of any underlying malignancies.

**Table 3:** Further pancreatitis work-up LDL: low-density lipoprotein; IgG: immunoglobulin G; PEth: phosphatidylethanol

Test	Result	Reference range
Cholesterol	120 mg/dL	<200 mg/dL
Triglycerides	130 mg/dL	<200 mg/dL
LDL cholesterol	41 mg/dL	<130 mg/dL
Calcium	9.3 mg/dL	8.2-10.2 mg/dL
IgG	1,165 mg/dL	700-1,600 mg/dL
PEth	Negative	Negative

## Discussion

This clinical case presents a rare cause of AP that is drug-induced and specifically caused by the class of thiazides. Drug-induced pancreatitis makes up only 2-5% of all pancreatitis cases [4], with the other more common causes being gallstones and alcohol use. The exact underlying mechanism of how drugs would induce the inflammation of the pancreas and cause it to present identically to gallstone or alcohol-induced pancreatitis is not yet understood. However, there are currently 500 medications known to cause AP [6], but only three medications caused AP in randomized controlled studies [7]. Although drug-induced pancreatitis is rare, a high index of clinical suspicion is important in identifying the possible agent as withdrawal of the agent is the definitive treatment [8].

AP is the inflammation of the pancreas with a wide spectrum of clinical severity ranging from mild self-limiting episodes to severe necrotizing cases with high mortality and morbidity [9]. Diagnosis of AP requires two of the following three criteria: abdominal pain consistent with the disease, serum amylase/lipase that is three times higher than the upper limit or normal, and characteristic findings of abdominal imaging [10]. However, meeting these criteria does not determine the etiology. Drug-induced pancreatitis is a diagnosis of exclusion after ruling out other common causes such as gallstones, alcohol use, and metabolic causes. It is then confirmed by the resolution of symptoms after the cessation of the offending agent [8].

In our case, gallstone was ruled out by an abdominal ultrasound that revealed no gallstones. Alcohol use was also ruled out through history-taking when the patient revealed she never drank alcohol throughout her life, which was confirmed by a negative PEth laboratory test. Similarly, hyperglycemia and hypercalcemia were ruled out as possible causes by normal serum calcium and triglyceride levels. Repeat abdominal imaging was necessary to confirm that the underlying malignancy was not a causative agent. This had to be done 4-6 weeks after the episode of AP to allow time for the inflammation to subside and ensure that malignancies would not be masked. The repeat abdominal CT scan was normal. Accordingly, the diagnosis of thiazide-induced pancreatitis was established by exclusion.

For such criteria of diagnosis, it is important to be aware of the common agents of drug-induced pancreatitis. A systematic review of the reports in the US Food and Drug Administration (FDA) Adverse Event Reporting System (FAERS) shows 62,206 reported cases of drug-induced pancreatitis between 2002 and 2024. The medication with the highest number of reported cases was metformin. It was then followed by quetiapine, liraglutide, exenatide, and sitagliptin [11]. In our case, the only known offending agent was hydrochlorothiazide with only 16 reported cases to this date [12]. The exact mechanism in which this class of drugs induces AP is not yet understood, but proposed mechanisms include direct pancreatic toxicity, ischemia due to reduced perfusion, and metabolic effects (e.g., hypercalcemia, hypertriglyceridemia) [13]. Direct pancreatic toxicity and ischemia due to reduced perfusion are more likely in our patient as the serum calcium and triglyceride lab values were normal.

Despite that, thiazides are one of the first-line medications used for hypertension [14]. They are effective, safe, and cost-efficient. They are unique within the group of first-line anti-hypertensive medications by reducing edema, which is a common adverse effect many patients complain from other first-line agents [15]. This makes thiazides a commonly prescribed medication accounting for 20-25% of hypertension prescriptions in the United States [16]. Hence, this case of a rare adverse effect should not deter clinicians from prescribing thiazides for hypertension but rather emphasize a possible adverse effect that requires a high index of suspicion.

Regardless of the underlying etiology, fluid resuscitation and supportive care are the gold standard of treatment for AP. However, identifying the underlying cause is important for the cessation of inflammation and prevention of recurrence. In our case, a thorough medication reconciliation and a high index of suspicion allowed for the early identification of the culprit with no recurrence of any episodes of pancreatitis for the patient as of today.

## Conclusions

This case explores the importance of recognizing drug-induced pancreatitis as a potential diagnosis in patients who present with AP in the absence of more common causes such as gallstones, alcohol use, or metabolic disorders. Although thiazides are effective, safe, and widely prescribed, clinicians should recognize their rare but potential trigger for AP. Early identification and discontinuation of the offending agent are essential to improve clinical outcomes and to reduce the risk of recurrence. Clinicians should maintain vigilance for this rare association, particularly when managing patients on thiazide diuretics, and ensure appropriate counseling and documentation to prevent further re-exposure.
